# Copra Meal: A Review of Its Production, Properties, and Prospects

**DOI:** 10.3390/ani14111689

**Published:** 2024-06-05

**Authors:** Jan Kathleen M. Punzalan, Kurt A. Rosentrater

**Affiliations:** Department of Agricultural and Biosystems Engineering, Iowa State University, Ames, IA 50011, USA

**Keywords:** copra meal, coconut, ruminants, feed ingredients, value added

## Abstract

**Simple Summary:**

There has been a growing focus on the utilization of copra meal, the byproduct of coconut oil production, as an alternative feed ingredient to supplement the growing demands of the ruminant livestock, poultry, and aquaculture industries. It provides a locally available and inexpensive source of protein, which is essential in tropical and subtropical regions, where coconut production is robust while conventional feed ingredients are scarce and prices are often unstable. This paper presents up-to-date information on the advancements in using copra meal as an animal feed ingredient and the various methods employed to enhance its nutritional and feeding value. It also offers a comprehensive overview of the physical and chemical properties of copra meal, emphasizing the need for further research to fully exploit its potential due to the limited understanding of its physical properties and the variability in quality resulting from different processing operations. This review paper also focuses on other potential applications for copra meal, which is crucial given the substantial global production volume with limited value addition. This could play a significant role in boosting the profitability of the coconut sector, reducing agricultural waste, and promoting sustainable agricultural practices.

**Abstract:**

Copra meal, the byproduct of coconut oil production, has been widely available at low cost but has been underutilized, with huge portions still becoming waste. Extensive research on different species have been performed to improve its use as an alternative feed ingredient, aiming to reduce the impact of fluctuating feed prices in some parts of the world where coconut is a major commodity. As for any biological product, the physical and chemical properties of copra meal play a crucial role in its use and limitations. In the case of copra meal, studies have found that additional treatments are needed to improve its nutritional composition and make it readily and efficiently available for ruminant and monogastric animals, poultry, and aquaculture applications. This paper presents a summary of up-to-date information on the physical and chemical characteristics of the product, as well as discussions on the various methods employed to improve and optimize its biological value as animal feed. There have been limited studies that have explored other effective and economical means of utilizing copra meal outside the livestock and feed industry. Hence, this paper also aims to provide a lens on future prospects and diverse applications involving copra meal, as well as to present the gaps and challenges that have to be addressed to maximize its product value and biological potential.

## 1. Introduction

In recent years, the search for a cost-effective and sustainable feed ingredient has led to growing attention towards the use of traditional resources, particularly in the tropical and subtropical regions of the world, where current growth in the agriculture and aquaculture industry is hampered by the geographical limitations in supply and fluctuating prices of conventional feedstuffs in the global market [1,2,3]. Feed expenses represent the largest overall operating costs, comprising about 50 to 80 percent for livestock, poultry, and aquaculture operations [4,5].

Among the residual byproducts of the oil industry that have been commonly used as an additive and partial substitute to feeds is copra meal. The increasing demand for coconut oil in the global market leads to significant quantities of copra meal produced as a byproduct of coconut oil production, with a recorded production volume of 1.98 million metric tons (MMT) in 2023, based on the latest data from the US Department of Agriculture (USDA) [6]. The remaining oil content offers an inexpensive source of energy and protein for animals; however, its use at high dietary levels is challenged by the poor amino acid profile and presence of anti-nutritional factors [7,8]. Extensive studies have been performed to overcome palatability issues and improve its nutritional and feeding value in different species through processing treatments and enzymatic supplementation, as well as to determine the optimum inclusion level without affecting animal performance [8,9,10,11,12,13,14,15,16,17,18,19,20,21,22].

Despite its significant volume and wide use, overviews are limited to the physical and chemical properties attributable to copra meal prior to post-processing operations to develop and utilize it in animal feeds and for other purposes. Hence, this paper aims to provide a review and summary of up-to-date information regarding its production, properties, and recent advancements in its use as a feed ingredient and in other agro-industrial applications. Such characterization is needed as a basis for value-adding activities and the optimization of the product given the growing demand for the utilization of copra meal as an alternative feed on a large-scale basis in key production areas in the world.

## 2. Materials and Methods

To ensure a comprehensive literature review, an extensive search of publications and previous studies dealing with copra meal, its chemical and physical properties, its use as an alternative animal feed ingredient for different agriculturally valuable species, and its value-added applications was conducted across multiple databases, including Google Scholar, PubMed, and Scopus. Keywords connected to coconut byproducts and other oilseeds was also included to ensure that potentially relevant articles were captured. Given the limited studies dealing with the characterization of copra meal, the inclusion criteria did not specify any given year as the review aimed to capture as much information as possible on this topic. However, studies involving the inclusion of copra meal as a substitute to traditional protein sources tested during feed trials highlighted publications from 1990 to 2023 only to cover recent developments in this area. Meanwhile, databases and reports from the federal government, multilateral agencies, and trade organizations relating to the industry situation and market trends of the coconut sector were also utilized.

## 3. Industry Situation

Coconut, dubbed the “tree of life” for its multitude of uses, ranging from the food industry, pharma- and nutraceuticals, cosmetics, and industrial and engineering applications, is widely produced in the tropical and subtropical parts of the world.

**Figure 1:**
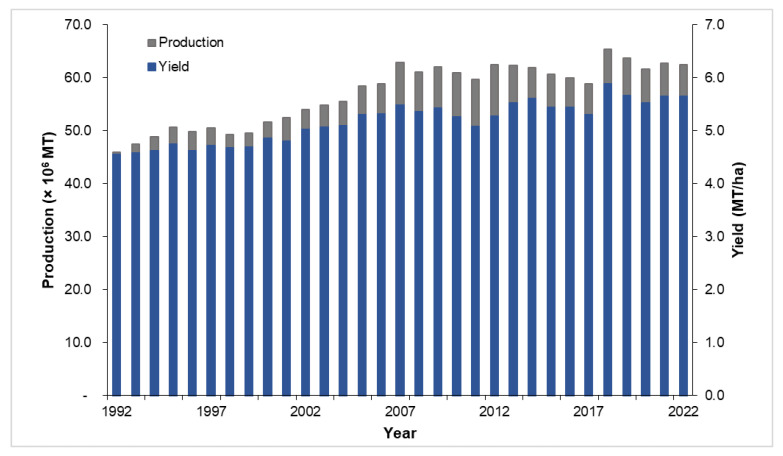
Global coconut production and yield [25].

With the significant increase in the worldwide supply and demand for coconut products and co-products over the past few decades, the coconut industry has played an important role in the economies of producer countries [23,24]. Coconut production reached a total of 62.41 million metric tons (MMT) in 2022, derived from the 11.05 million hectares devoted to coconut plantation, based on the latest data from the Food and Agriculture Organization (FAO) [25]. Figure 1 shows the 20-year global production and yield of coconut, with peak values occurring in 2018. There was a slight decrease observed in the overall production in 2019 and 2020, while the 2021 production showed an increase of 2.1% as compared to the previous year. Despite the relatively flat trend in the overall yield recorded in 2022, the global coconut production is forecasted to reach 65.4 MMT by 2026, exhibiting a conservative 0.5% year-on-year growth from its 2021 levels [26].

The bulk of the production was derived from Asia and the Pacific Region, comprising 88.7% of the production share. Major producing countries recorded in 2022 included Indonesia (17.19 MMT), the Philippines (14.93 MMT), and India (13.32 MMT), while the consumption and use of coconut is dispersed throughout the globe [25].

## 4. Market Trends and Supply Projections

Coconut and its byproducts are highly traded commodities in the global market. More than 50 different forms of coconut products, including unprocessed, semi-processed, or processed co-products and byproducts, are entering the international market in significant quantities. Aside from coconut oil, copra, and fresh coconut, dominating the global trade, there is an increasing demand for other coconut products, including coconut coir, fiber, desiccated coconut, coconut water, coconut sugar, coconut milk, and copra oil cake and other solids [27,28,29].

The total market share of coconut co-products and byproducts was estimated at USD 20.24 billion in 2022. The industry is forecasted to grow at a compound annual growth rate (CAGR) of 8.4% from 2023 to 2030. One factor seen in the growing demand for coconut-based products is the increasing attention to health and preferences for plant-based diets, especially in the context of the recent COVID-19 pandemic [30,31].

The International Coconut Community (ICC), an intergovernmental organization established in 1969 under the United Nations Economic and Social Commission for Asia and the Pacific (UN-ESCAP), serves as the peak industry body for the development of the coconut industry through its member countries. The organization consists of 20 major coconut-producing countries around the globe, which account for more than 90 percent of the world’s total coconut production and trade [27].

The latest statistics from the ICC on the global trade of coconut oil for the same period of 2021 and 2022 shows a robust demand for coconut oil, with a 17.4% overall increase worldwide as indicated in Table 1. This is despite the movement restrictions in the last two years brought about by lockdowns during the pandemic and post-pandemic era and the tensions since 2021 caused by the Ukraine–Russia war. The global trade of coconut oil is expected to be stable, with the majority of the supply sourced from the Philippines’ and Indonesia’s production and exports.

**Figure 2:**
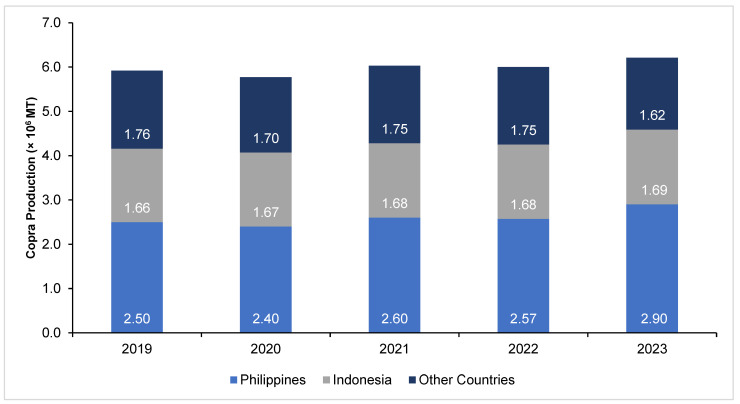
Global copra production [6,32,33,34,35,36,37,38].

As the main input of coconut oil production, the supply of copra is directly related to the stable outlook of the coconut oil industry. The global copra production reached 6.21 million metric tons (MMT) in 2023, based on the latest report of the United States Department of Agriculture (USDA), a 3.5% increase from the 2022 level of 6.0 MMT. The Philippines and Indonesia remained as the top copra producers, as shown in Figure 2, with recorded production of 2.90 MMT and 1.69 MMT, respectively [6].

Meanwhile, copra meal, which is the byproduct of coconut oil production, recorded a trade value of USD 160 million in 2021 alone, at an export growth rate of 38.4%. The top exporters are the Philippines, Indonesia, and Sri Lanka, while the bulk of the imports are from India, South Korea, and China. In 2018, the mean tariff rate for copra was 7.62%, while that for copra meal was 5.56% [28].

The trade of coconut oil and its byproducts in the international market are governed by the set standards of the National Institute of Oilseed Production (NIOP), the Federation of Oilseed, Fats and Oils (FOSFA), and the ASEAN Vegetable Oil Club (AVOC). Among the parameters used to classify the grades and standards of copra are the oil content, moisture content, and appearance and color of the product [39]. Additional requirements are set depending on the exporting country, such as those outlined in IS 6220-1971 standard [40] of India and in PNS/BAFS 43:2009 standard [41] of the Philippines. Other restrictions are also imposed by the importing countries, including the limit for aflatoxin regulated in the EU region [42]. It is crucial to attain the standard quality and grades of copra required for domestic use and export, since the prices and market access vary significantly based on these parameters.

## 5. Copra Meal Production

One of the main traditional products derived from coconut is copra, which refers to the fresh coconut meat dried to low moisture content ranging from 6 to 8% to concentrate the oil content and ensure safe storage. The quality of the copra, coconut oil, and copra meal byproduct is highly influenced by the maturity of the nuts used, the drying method, and storage. The processing of immature nuts could result in rubbery copra with high moisture content, while overripe ones result in thinner copra with lower oil content. Meanwhile, the improper drying and storage of copra often lead to an increased incidence of aflatoxin-related molds due to high moisture content, which accounts for the majority of postharvest losses in the coconut oil and copra supply chain [42].

The cheapest and most commonly used drying method employed for copra production includes sun and kiln drying, but the longer drying time, risks of fungal and aflatoxin contamination, and variable quality of copra make it more convenient to utilize hot air using biomass or fuel-driven mechanical dryers [43,44,45]. Various studies on the use of solar-powered methods of drying, such as forced convection solar dryers and solar tunnel dryers, to economically produce high-quality copra show that these have also become popular in recent years [46,47].

Crushed copra is then used as an input for the dry process of coconut oil production. Note that oil production from coconut can be categorized into a wet or dry process depending on the starting material, with the wet process utilizing undried coconut meat subsequently processed into a coconut milk emulsion and the dry process involving oil extraction using copra. Details of the wet method are outlined in Ng et al. [48] and Divya et al. [49], while an in-depth discussion of the step-by-step procedures of the dry process, both of which are beyond the scope of this paper, can be found in Punchihewa and Arancon [42], Seneviratne and Jayathilaka [45], and the World Bank report [50].

Focusing on the dry method, coconut oil can be extracted either mechanically, using an expeller or hydraulic presser, or through solvent extraction. The mechanical extraction of dried copra is usually preferred for its cost-efficiency and the safe recovery of the oil. Extraction using organic solvents, typically n-hexane, yields lower oil recovery and may affect the quality of the oil due to the residual solvent and high temperatures [43,45,51]. The method also poses some environmental concerns and human hazards since n-hexane is known to have flammable and explosive properties [45]. Moreso, a study by Lee and Kim [51] revealed that mechanically extracted CM tends to have greater ether content as compared to solvent-extracted ones. On the other hand, studies by Thomas and Scott [52] suggest that solvent extraction results in CM with improved quality due to the fact that the essential amino acid, lysine, tends to be more severely destroyed during mechanical extraction.

**Figure 3:**
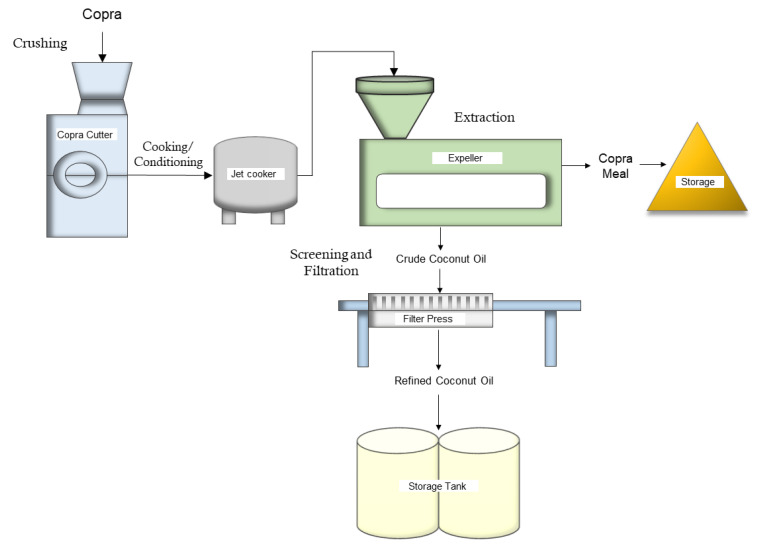
General process flow of coconut oil production via mechanical extraction.

The general process flow of mechanically extracting copra to produce coconut oil is illustrated in Figure 3. The resulting byproduct of coconut oil production is copra meal (CM), interchangeably referred to as “copra cake” or “de-oiled copra”. The remaining oil content in the product, which amounts to approximately 7% oil depending on the extraction method used, provides a valuable energy source to supplement expensive imported feeds in areas where conventional feed ingredients are limited. On average, five coconuts are needed to produce 1 kg of copra. Each kilogram of copra will then yield 610 g of oil and 370 g of copra meal [50].

## 6. Copra Meal as an Animal Feed Ingredient

In the Asia–Pacific Regions, where there is the bulk production of coconut products and byproducts, the use of copra meal as an alternative feed ingredient is economically advantageous. It is locally available and provides an inexpensive source of protein and energy. However, there are a considerable number of limitations in the use of copra meal (CM) for animal feed, mostly associated with its physicochemical and nutritional characteristics.

Copra meal has long been used for ruminant feeding but has limited applications in monogastric, poultry, and aquaculture diets due to its relatively high crude fiber content [42,53]. Several attempts have been made to address these limitations in the nutritional properties and feeding value through improving the efficiency and quality of the protein extraction of copra meal or by changing its composition through various methods, including physical alterations, enzymatic treatments, and fermentation. Key studies conducted on the use and development of copra meal as a feed ingredient for agriculturally valuable species are summarized in Table 2.

In an effort to establish the extent at which the fiber content of CM affects its nutritional value and protein usage in animal diets, Lachance and Molina [53] devised experiments to analyze the amino acid content and protein score of a fiber-free coconut protein extract obtained through an enzymatic chemical method and compared it with a commercially produced CM. The results revealed the consistently superior biological value of proteins with a higher amino acid composition for fiber-free extracts as compared to regular CM, which is also in line with the previous findings of Rao et al. [54], indicating decreased protein quality with increased fiber content.

Meanwhile, Sulabo et al. [55] suggest that the lower-quality amino acids in copra are likely a consequence of heat damage during the drying and oil extraction process. Jaworski et al. [11] specifically tested experimental diets using copra meal on weanling pigs. It was found that a high concentration of fiber, specifically soluble dietary fiber, in CM is a deterrent factor regarding feed intake in pigs. Previous findings by O’Doherty and McKeon [12], who focused on the inclusion of CM in the diets of grower and finisher pigs, also supported these results, finding that the depressive effects of a high NDF composition in CM were more prominent in growing pigs than in finishing pigs due to their more developed hind gut fermentation. As expected, higher concentrations of CM in the swine diet resulted in decreased organic matter, protein, and energy digestibility, but the experiments also yielded an improved food conversion ratio, leading to potential areas for development in the formulation of CM to make it more effective in swine feeding.

The potential incorporation of copra meal in aquaculture feed diets was also tested in various species, particularly in Nile and saline tilapia, milkfish, and black tiger shrimp, in the studies conducted by Obirikorang et al. [15], Harlina et al. [14], Magbanua and Ragaza [56], Apines-Amar et al. [13], and Corre et al. [17], respectively. Similar to the trend observed for other experimental diets for animals using copra meal, the inclusion of CM resulted in lower feed bulk density. Note that the physical properties of feed, specifically its bulk density, are crucial in the sinking rates, water stability, and nutrient retention efficiency of feed pellets in aquaculture applications. A higher concentration of copra meal resulted in negative effects on fish performance; however, it was notable that the proximate composition, with the exception ash, of tilapia carcasses fed a CM diet was not statistically different from that of fish fed using a fish meal-based diet, attributed to the efficiency of the crude protein, dry matter, and energy content of the product.

The results of the experiments conducted to test the effectiveness and extent of the partial replacement of conventional feed ingredients with copra meal also revealed the limitations of the use of CM due to its chemical composition and inherent properties. However, the notable results provide a significant opportunity to develop a cheap, valuable, and readily available source of dietary energy and protein for livestock, poultry, and aquaculture, given that a careful and optimal diet is formulated.

As mentioned, one of the constraints on the use of CM as a feed ingredient is the presence of anti-nutritional factors, particularly tannin, which hinder the growth of animals and are even found to be toxic in fish. The effect of varying levels of treated (soaked in tap water at room temperature for 16 h) and untreated CM in formulated feed diets in terms of the proximate composition, feed utilization, and protein digestibility of CM was tested by Mukhopadhyay and Ray [8] on carp fingerlings. Water treatment was found to reduce the tannin content by 0.9%, resulting in increased CM digestibility.

The effect of fermentation to improve the feeding value of CM was also tested in laying hens in Dairo and Fasuyi [9] and in saline tilapia (*Oreochromis niloticus*) seeds in Harlina et al. [14]. The fermentation process resulted in increased levels of crude protein, an improved amino acid profile, and lower crude fiber content, which resulted in a higher body weight gain and egg production for laying hens. Meanwhile, fermented CM led to a higher digestibility level when tested in tilapia as compared to untreated samples. The additional fermentation step breaks down some of the anti-nutritional content of CM, including the high dietary fiber.

The inclusion of copra meal in animal diets has shown variable effects on the overall performance and growth of species across different studies. In poultry applications, the use of copra meal as an alternative to cereal grains may be limited due to poor growth performance at high inclusion levels. However, enzyme treatments and fermentation are shown to minimize the effects on performance characteristics and production due to the improved amino acid profile [9,10]. Pelleting and crumbling to increase the bulk density of CM-based diet also led to improved body weight in broilers [7]. Meanwhile, the use of CM as an alternative feed ingredient was found to maintain the feed efficiency, digestibility, and carcass performance in pigs, with optimal supplementation varying from 15 to 20% [11,12]. In aquaculture, the use of copra meal is mainly driven by the reduction in feed costs when supplementing traditional feed ingredients such as soybean meal and fish meal. Generally, CM is deemed to be more beneficial when included in the diets of herbivore and omnivore fish species as opposed to carnivorous ones [1], although treatments such as water soaking and fermentation to improve digestibility and properties are found to enhance inclusion levels regardless of the species’ diets [8,13,14,15,16,17]. No significant adverse effects were found in terms of the survival rates, feed conversion efficiency, and carcass composition when reformulating diets in terms of the protein source at appropriate inclusion levels, as listed in Table 2. The high digestibility of copra meal, particularly due to the low lignification of its cell walls, suggests that it can be an efficient source of energy, supporting good growth rates in ruminants [18,19,20]. Moreover, the provision of copra meal has been associated with improvements in milk production and quality in dairy cows, as well as enhanced growth rates in beef cattle, indicating its utility as a valuable supplement in ruminant nutrition [20,57]. However, the variability in the responses highlights the need for the careful consideration of copra meal’s inclusion rate and the overall composition of the diet, so as to maximize its benefits while mitigating any potential drawbacks related to its palatability and feed intake [43]. In the realm of sustainability, incorporating coconut-based products, such as CM and coconut oil, was found to reduce the methane and carbon dioxide output from ruminants, deeming them valuable in reducing greenhouse gas emissions from the livestock industry [19,21]. There has also been a specific focus on utilizing CM as a feed component in equine diets due to its low nonstructural carbohydrate (NSC) levels, which makes it appropriate for horses with metabolic issues that hinder the processing of starch and sugar [22].

Overall, the inclusion of copra meal in animal feeding can serve as a viable alternative in the diets of various species that produces results comparable to those of other conventional feed ingredients. However, optimization of treatment processes and the strategic formulation of diets is needed to improve its feeding value and further increase optimal inclusion rate. Processing techniques, such as fermentation and enzyme supplementation, combined with tailored diet formulation based on the nutrient requirements specific for the species, could significantly improve the amino acid profile, palatability, and digestibility of copra meal. The nutrient uptake and retention of CM-based diets can also be enhanced through manufacturing processes, such as pelleting and extrusion, as it improves its physical properties. This, in turn, would make CM a more cost-effective feed ingredient that can be used to supplement cereal grains and other traditional energy and protein sources for animal feeding in regions where there is wide availability of coconut, while conventional feed ingredients are expensive and have limited availability.

**Table 2:** Summary of findings on the use of copra meal as a feed ingredient for agriculturally valuable species.

Species	Experimental Conditions	Key Results	Reference
Broilers	**Control:** Control corn–soybean diet given from day 1 to day 4	Significant linear decrease in feed intake (339.0 to 250.4 g; 0% to 50% CM inclusion), weight gain (300.1 g to 148.5 g), FCR (1.13 to 1.72), DM digestibility (80.1% to 64.0%), and AME (13.33 MJ/kg to 12.21 MJ/kg) with increasing levels of CM. Addition of enzyme treatment improved all parameters, except feed intake.	[10]
	**Experimental:** Four basal diets (0, 10, 30, and 50% CM) and four enzyme treatments to break down main polysaccharide components (0, Hemicell^®^, Allzyme SSF^®^ or a mixture of Hemicell, Gamanase^®^, and Allzyme SSF)		
	**Parameters measured:** Mean feed intake, weight gain, FCR and DM digestibility, nutrient digestibility, apparent metabolizable energy of the diets, and jejunal viscosity		
Laying Hens	**Control:** Control diet of soybean meal (SBM)	Optimal replacement of 50% soybean meal (SBM) using fermented CM protein with minimal effects on the performance of laying hens. Feed intake varied from 120.64 g to 124.96 g from 0% to 75% SBM replacement, from 2.07 feed/kg eff to 2.18 in terms of feed efficiency, from 72.42% to 68.09% in terms of hen-day production, and from 1.78 ₦ ^1^/kg to 1.74 ₦ ^1^/kg for feed cost per egg. Similar values were obtained for body weight at 1.58 g.	[9]
	**Experimental:** Experimental diets of fermented copra meal (CM) as substitute to SBM at 0, 25, 50, and 75% level based on protein content		
	**Parameters measured:** Performance characteristics (i.e., feed intake, feed efficiency, hen-day production, body weight, feed cost per egg) and hematological indices of egg		
Weanling Pigs	**Control:** Control diet containing corn, SBM, and 4% fish meal	Up to 15% supplementation if diets are formulated based on digestible nutrients and ME, with no significant effect on gain to feed ratio (from 0.67 in the control diet to 0.64 at 15% CM inclusion) but potential decrease in overall ADG (from 512 g/day to 464 g/day) and ADFI (from 765 g/day to 721 g/day).	[11]
	**Experimental:** Experimental diets formulated with 5, 10, and 15% CM substituted for corn and SBM		
	**Parameters measured:** Average daily gain (ADG), average daily feed intake (ADFI), and feed efficiency (G:F)		
Grower and Finisher Pigs	**Control:** Control diet using barley and 0% CM	20% CM inclusion in grower–finisher pig diets resulted in mean digestibility coefficients of 87.9 for organic matter, 84.6 for protein, and 85.5 for energy. The values tended to decrease at increased levels of CM. Diets formulated with 20% CM on a least-cost basis exhibited an increase in the live weight gain from 0.886 kg/day to 0.897 kg/day, from 2.63 to 2.48 FCR, and from 734.0 g/kg to 713.0 g/kg in terms of kill-out proportion. Overall, 20% replacement of barley with CM formulated on a least-cost basis had an insignificant effect on the overall performance in the combined grower–finisher phase of pigs.	[12]
	**Experimental:** Experimental diets at varying levels of CM (20 and 40% for digestibility experiment; 10 and 20% as direct replacement for barley and formulated on a least-cost basis for performance experiment)		
	**Parameters measured:** Nutrient (OM, protein, energy) digestibility; carcass performance (growth rate and kill-out proportion)		
Indian Major Carp	**Control:** Control diet with fish meal as the main protein source	20–30% fish meal replacement using treated CM (to reduce tannin content). Weight gain varies from 73.68% using the control diet to 83.58% using treated CM, feed intake from 1.53 g/day to 1.50 g/day, SGR from 0.919 %/day to 1.01 %/day, and FCR from 2.27 to 1.94.	[8]
	**Experimental:** Untreated and treated (soaked in tap water at room temperature for 16 h) raw copra meal was incorporated in the experimental diets at 20, 30, and 40% fish meal replacement by weight		
	**Parameters measured:** Growth performance, feed utilization efficiency, and carcass composition		
Milkfish	**Control:** Control diet with SBM as main protein source	Optimal inclusion of 5% fermented CM equivalent to 12% SBM protein replacement for superior growth and FCR. Feeding trial in milkfish using fermented CM yield 0.69 FCR, 3.70% SGR/day, and 100% survival within 35-day culture period. Proximate composition of fish carcass at 5% FCM inclusion also produced comparable results with the SBM-based control diet, with carcass having mean crude protein of 56.5%, 34.0% crude fat, 0.04% crude fiber, 7.55% ash, and 3.45% NFE on a dry matter basis.	[13]
	**Experimental:** Diets formulated containing 0, 5, 10, 15, 20, and 25% fermented CM as partial replacement to SBM protein		
	**Parameters measured:** Specific growth rate (SGR) and survival, feed conversion ratio (FCR), and proximate composition of fish carcass		
Saline Tilapia	**Control:** Feed treatment with 0% CM inclusion and fish meal as main source of protein	15% optimal level of inclusion of fermented copra meal with better total feed digestibility (48.80%) and improved protein content (36.65%).	[14]
	**Experimental:** Varying concentrations of fermented, dried, and powdered copra meal (15, 30, 45%) were incorporated to the feed treatments as plant-based source of protein		
	**Parameters measured:** Feed digestibility and composition		
Nile Tilapia	**Control:** Control diet with fish meal as main protein source	Potential inclusion of 30% unrefined CM with no negative effects on feed intake. Results yielded reduced feed bulk density of 344.26 g/L and mean sinking velocity of 7.13 cm/s. Higher feed intake and fecal production was recorded at 283.10 g and 372.6 g DM/kg ingested feed, respectively.	[15]
	**Experimental:** Inclusion of 30% CM in the experimental diet		
	**Parameters measured:** Feed bulk density, sinking velocity, feed intake, fecal production		
Grouper	**Control:** Fish meal and soybean meal-based diet with 0% fermented copra meal replacement	Optimal SBM replacement of up to 100% (16% in diet) using fermented copra meal without significant adverse effects on fish performance and carcass composition. Results yielded 71.1% survival rate, 0.57 FCE, 4.64 g/day feed intake, and 974% weight gain during the 70-day feeding trial.	[16]
	**Experimental:** Replacement of soybean meal (SBM) with fermented copra meal at varying concentrations (25, 50, 75, 100%) with and without amino acid (methionine and lysine) supplementation		
	**Parameters measured:** Survival rates, feed conversion efficiency (FCE), feed intake, weight gain, carcass composition		
Black Tiger Shrimp	**Control:** Basal shrimp diet using fish meal as primary source of protein	Up to 40% replacement for fish meal protein without significant detrimental effect on growth (SGR = 2.2 %/day), survival (81.8%), and feed efficiency (FCR = 2.1).	[17]
	**Experimental:** Fermented CM-based diet replacing fish meal protein at varying levels (0, 10, 20, 30, and 40%) of the diet		
	**Parameters measured:** Specific growth rate (SGR), percent survival, feed conversion ratio (FCR)		
Goats	**Control:** Control diet using soybean meal (SBM) as primary source of protein and 0% copra meal (CM) included	Up to 50% SBM substitution with CM resulted in comparable performance of goats in terms of feed intake (from 71.4 g/day to 73.7 g/day; at 0 to 50% diet replacement), apparent digestibility (57.7% in terms of dry matter, 62.4% in terms of organic matter, 56.1% in terms of crude protein, and 50.7% in terms of neutral detergent fiber at 50% CM replacement), and body weight gain (from 60.0 g/day to 62.5 g/day; from 0 to 50% diet substitution).	[18]
	**Experimental:** Concentrate mixtures consisted of copra meal (CM) at varying levels (25%, 50%, 75%) as replacement for dietary crude protein provided by SBM		
	**Parameters measured:** Feed intake, apparent digestibility, live weight change		
Sheep	**Control:** Diet consisted of alfalfa hay, corn stover, corn grain, ground sorgum, soybean meal, cane molasses, urea, and 0% copra meal (CM) forumulated based on the nutritional requirements for lambs	Similar growth performance at an average daily gain of 0.23 g/day was observed in lambs fed with CM-based diets. Feed conversion increased from 5.48 to an average of 6.1 for the three treatments. Meanwhile, the gas production volume, particularly that of methane and carbon dioxide, tended to decrease among treatments with CM, with the lowest recorded gas production volume of 153.2 mL/g at a 150 g CM level. This suggests the potential role of utilizing CM-based diets in reducing greenhouse gases emissions from the livestock industry.	[19]
	**Experimental:** Treatments consisted of CM at 50, 100, and 150 g/kg DM, along with the same ingredients in the control diet and similarly formulated based on the nutritional requirements for lambs		
	**Parameters measured:** Average daily gain, feed conversion, gas production rate		
Cattle	**Control:** Basal diet of *Imperata cylindrica* native pasture and mixed legumes (less than 20%)	Supplementation of CM in the diet of Brahman weaner steers (young male cattle) improved the live weight gain by 96 g/day. Further addition of molasses and urea to CM-supplemented diet increased the body weight gain by 159 g/day.	[20]
	**Experimental:** Diet was supplemented with 2/3 CM for bypass protein. Additional experimental diet containing 1/3 molasses for rumen fermentable energy and urea (±3%) for rumen degradable nitrogen was also tested		
	**Parameters measured:** Live weight gain		
Dairy Cows	**Control:** Tropical pasture with no supplement	Milk yield increased from 12.4 kg/day without supplement to 13.2 kg/day and 12.7 kg/day for 3 and 6 kg/day CM inclusion, respectively. A significant increase of up to 15.8% in milk fat content was also observed. Rumen pH was maintained at 7.1 when CM was added. Reduced live weight was also recorded for supplemented dairy cows at an average of 5 kg during the 12-week trial.	[57]
	**Experimental:** Copra meal incorporated as supplement to tropical pasture at 3 kg/day and 6 kg/day levels		
	**Parameters measured:** Milk yield, composition, rumen pH, live weight		
Horses	**Control:** Pasture-only control diet consisted of 90% *Pennisetum clandestinum* and 10% *Trifolium repens*, with approximately 7% non-structural carbohydrate (NSC) content on a dry matter (DM) basis	Average body weight was maintained over the 25-day feeding trial at an average of 456 kg. CM-based treatment obtained the lowest post-feeding plasma glucose level of 4.4 nM/L, comparable to the 4.2 nM/L peak glucose level of the control diet.	[22]
	**Experimental:** Pasture supplemented with copra meal (CM) with approximately 11% DM NSC content, pelleted feed (25.3% DM NSC), and pre-mixed sweetfeed (33.7% DM NSC)		
	**Parameters measured:** Body weight, plasma glucose response		

^1^—₦ 128 = USD 1.

## 7. Properties

As discussed in the previous section, existing studies on copra meal have focused on its development as a partial substitute and additive for animal feeds. Various publications provide discussions on the proximate analysis of copra meal, as summarized in Table 3, and how these can be changed through various methods to improve the digestibility and feeding efficiency. While studies have dealt with the chemical and nutritional properties of copra meal (CM) as a foundation for the improvement of its biological value, there are still gaps in the physical characterization of this product that have yet to be understood to obtain a clearer picture of the interplay of all of its properties in its application, development, and value addition.

### 7.1. Chemical Properties

Copra meal consists of relatively high carbohydrate and protein content, as shown in Table 3. However, a significant amount of the total carbohydrate is regarded as unusable fiber, which mainly consists of non-starch polysaccharides (NSP), generally water-insoluble non-cellulosic polysaccharides [58]. The predominant NSP in CM is mannan, while traces of cellulose and galactomannan are also found in significant portions [7].

The nutritional analysis of CM also yields relatively low lysine content, indicating this as the probable limiting amino acid [53], while others, such as methionine, tryptophan, and threonine, are deemed insufficient to meet the specifications for feeding applications in monogastric animals [3,59,60]. These findings indicate that the use of CM as a protein source is hindered by its low usable energy value, caused by the limited amino acid concentrations and high dietary fiber content. Although CM was found to have potentially higher gross energy concentrations than soybean meal and corn in the proximate analysis performed by Sulabo et al. [55], the elevated dietary fiber content leads to lower digestible and metabolizable energy. Note that crude fiber is the traditional measure of fiber content in feed but only accounts for most of the cellulose and some of the lignin components. On the other hand, neutral detergent fiber (NDF) measures the total amount of the plant cell wall, including cellulose, hemicellulose, and lignin, hence being more useful in the analysis of the feeding value of a plant-based product.

Copra meal also contains considerably higher levels of crude fat as compared to other edible oil cakes, such as soybean, rapeseed, cotton seed, and palm kernel [61]. This high fat content, composed of short-chain saturated fatty acids, is a result of the residual oil left in the copra meal after extraction. The fat content is an important parameter directly correlated to the heating value of a product. Moreover, as emphasized in the study conducted by Szyszlak-Bargłowicz et al. [62], the significant amount of fat in CM contributes to reducing the energy consumption when using it as an additive in the pelleting process, since fat serves as a lubricant that lowers the friction between and among the particles and the mill. Meanwhile, this inherent characteristic of CM leads to negative effects in terms of the durability of the product.

The chemical properties of the raw material greatly affect its inherent binding characteristics and will have significant effects on the quality and stability of feeds during applications [15]. Some studies also cover the analysis of the fecal quality of species as a function of the feed ingredient, as these solid wastes have direct correlations in assessing the environmental impact of the product and species within a cradle-to-grave scope.

Listed in the table below is a summary of the chemical properties of copra meal as found in several published papers that aimed to analyze and improve its use as a feed ingredient. It can be noted that significant variation exists in the summarized values of each property, as they are highly dependent on the origin of the raw material analyzed and the processing operations that the samples have been subjected to.

**Table 3:** Chemical properties of copra meal.

Property	Reported Values, % Dry Basis				References
	Minimum	Maximum	Mean	S.D.	
Dry Matter	87.10	92.90	90.17	2.22	[2,3,8,9,10,11,12,15,21,55,59,60,63,64]
Gross Energy (kCal/kg)	4371	4785	4603	163.41	[3,8,10,11,12,21,55,60,64]
Digestible Energy (kCal/kg)	3272	4071	3717	266.38	[3,11,22,55,60,64,65]
Metabolizable Energy (kCal/kg)	3110	3903	3554	251.74	[9,11,12,55,60,64,65,66]
Crude Protein	19.63	24.29	22.94	1.34	[2,3,8,9,10,11,12,15,21,22,53,55,59,60,63,64]
Arginine ^E^	2.13	3.54	2.53	0.54	[3,8,9,10,11,55,59,60,64]
Cysteine ^E^	0.27	0.32	0.30	0.02	[3,8,9,55,59,60,64]
Glycine ^E^	0.88	1.01	0.93	0.06	[3,9,55,59,60,64]
Histidine ^E^	0.38	0.78	0.45	0.14	[3,8,9,11,55,59,60,64]
Isoleucine ^E^	0.67	0.92	0.74	0.09	[3,8,9,11,55,59,60,64]
Leucine ^E^	1.27	1.48	1.35	0.08	[3,8,11,55,59,60,64]
Lysine ^E^	0.23	0.63	0.50	0.11	[3,8,9,10,11,12,55,59,60,64]
Methionine ^E^	0.29	0.46	0.36	0.06	[3,8,9,10,11,55,59,60,64]
Phenylalanine ^E^	0.46	0.95	0.83	0.16	[3,8,9,11,55,59,60,64]
Threonine ^E^	0.59	0.99	0.70	0.14	[3,8,9,11,55,59,60,64]
Tryptophan ^E^	0.13	0.21	0.16	0.03	[11,55,59,60,64]
Valine ^E^	0.92	1.16	1.03	0.07	[3,8,9,11,55,59,60,64]
Alanine	0.90	0.92	0.91	0.01	[3,9,55,60,64]
Proline	0.65	0.80	0.73	0.08	[3,9,55,60,64]
Tyrosine	0.14	0.63	0.45	0.15	[3,8,9,55,59,60,64]
Serine	0.76	1.07	0.90	0.13	[3,9,55,59,60,64]
Aspartate	1.61	1.84	1.72	0.11	[3,9,55,60,64]
Glutamate	3.60	4.08	3.87	0.25	[3,9,55,60,64]
Total Carbohydrates	45.89	47.35	46.62	1.03	[8,53]
Crude Fiber	6.60	18.21	13.04	3.59	[2,3,8,9,10,15,21,53,59]
Acid Detergent Fiber (ADF)	27.72	29.30	28.78	0.61	[11,21,55,60,64]
Neutral Detergent Fiber (NDF)	55.76	68.33	59.48	3.93	[10,11,12,21,55,60,63,64]
Crude Fat	2.28	16.14	9.00	4.05	[2,8,9,10,12,15,21,22,53,59,60,64]
Ash	5.05	9.10	6.88	1.11	[2,3,8,9,10,11,12,15,21,53,55,63]
Calcium	0.04	0.23	0.12	0.08	[3,11,22,59,60,63,64]
Phosphorous	0.50	0.71	0.59	0.07	[3,11,22,59,60,63,64]
Potassium	1.53	2.00	1.89	0.20	[3,22,59,60,64]
Chlorine	0.03	0.77	0.40	0.30	[22,59,60,64]
Magnesium	0.26	0.36	0.32	0.04	[3,22,59,60,64]
Sodium	0.04	0.04	0.04	0.002	[22,59,60,64]
Sulfur	0.33	0.34	0.34	0.002	[60,64]
Manganese (ppm)	58.70	83.43	72.85	10.31	[3,59,60,64]
Copper (ppm)	26.91	27.17	27.04	0.19	[60,64]
Iron (ppm)	523.14	528.26	525.70	3.62	[60,64]
Zinc (ppm)	52.74	58.95	54.99	3.45	[3,60,64]
Phytic acid	0.20	0.87	0.49	0.34	[8,11,60,63,64]
Tannin	2.40				[8]

^E^—essential amino acids.

### 7.2. Physical Properties

Only a few studies have focused on the physical properties of copra meal, listed in Table 4, and much of the information provided so far associates these characteristics with the product’s low feeding value in monogastric and poultry diets.

The hard and crystalline physical features of copra meal are associated with its very high mannose to galactose ratio. Its gritty appearance and coarse texture, due to the branched polysaccharides, predominantly the non-starch polysaccharide (NSP) mannan, as discussed in detail by Knudsen [58], has been found to limit its feeding value due to its tendency to increase the viscosity of the intestinal contents in monogastric animals. The high insoluble fiber content also affects the nutrition intake as well as the quality of litter in poultry applications [7,10].

A study conducted by Sundu et al. [10] revealed that the bulk density and water holding capacity (WHC) of copra meal greatly affect its quality as an animal feed, with even potentially greater effects than its chemical characteristics. The low bulk density and high WHC of CM lead to decreased feed intake in poultry species, which has detrimental effects on their growth rates. Altering the physical properties of CM through pelleting and crumbling to increase the bulk density resulted in a higher body weight and gizzard size in broilers as compared to enzymatically treated CM, and, surprisingly, even in comparison to a conventional corn–soy diet. Furthermore, a consequent study conducted by Sundu et al. [7] covered the effect of the average particle size of CM in poultry diets, indicating that a larger particle size yielded heavier and larger gizzards due to higher feed intake and the higher volume of the digesta. In aquaculture feeding, an increase in shear effects at high screw speeds during feed processing could lead to the mechanical destruction of starch molecules, thereby further increasing the WHC [67].

Aside from the bulk density being a key parameter in determining the quality of feed ingredients, it also plays an important role in the transport, handling, and storage efficiency of biological products. A higher bulk density increases the transport capacity and reduces the storage space, which have direct impacts on the logistical costs and crumbling-related losses in pelleted products [62].

These findings indicate a significant need to further understand the physical characteristics of CM—for instance, determining the required bulk density to reach the optimal growth of species in feeding applications and how this characteristic could possibly provide other opportunities and breakthroughs in other applications.

Another important area is the analysis of the color of copra meal, since this property is highly affected by the heat treatment and extraction method that the input material, the raw copra, was subjected to. Note that, for raw copra, the moisture content and appearance, indicated through the color, are used as the main parameters in determining the quality and grade of copra and therefore the price. Experiments quantifying the effect of varying drying air temperatures on these two characteristics were performed on unprocessed copra by Guarte et al. [68]. However, no study has been carried out to completely analyze the color of copra meal and consider this as a function of the processing operations performed and the ways in which it affects other chemical properties. The color has been associated with the nutritional value of a product and could provide an opportunity to understand the correlations between its other intrinsic properties, such as drying behavior and heat capacity [69]. In the experimental setup of Sundu et al. [10], the overheating of CM during drying or oil extraction, indicated through the very dark brown color of CM samples, was a potential factor for the low digestibility of CM-based diets. Maillard products could be developed, reducing the feed digestibility, along with the destruction of nutrients in the process.

Color and odor, associated with rancidity, are also indicators of the aflatoxin content of CM, which are known as limiting factors in the quality and acceptability of CM in the global market. These quality parameters are also known to affect the feed efficiency, as found in the trials conducted in swine by Schell et al. [70]. In terms of aquaculture feed applications, it would be critical to examine the physical characteristics, particularly the hardness, water stability, water absorption index, and water solubility index, as these are important indicators of the nutrient retention capacity and sinking velocity of feed pellets. The hardness of feeds is also highly correlated to the animal’s preference, especially in aquatic species, as studies have found that fish prefer softer pellets than harder ones and the high concentration of CM in experimental feed diets result in lower feed intake [56]. Note that the inclusion of plant ingredients in feed has significant effects on the physical properties of the product, since the NSPs have the potential to reduce the expansion and increase the hardness.

The storage conditions and durations of copra meal have also been proven to greatly affect the moisture content and odor of the product. The moisture content is known to significantly influence other physico-chemical properties of biological products. Looking into the effects of the varying moisture content of copra meal, and quantifying its effect and how it is correlated with the other measurable characteristics, is a promising area to pursue because it plays a crucial role in the potential uses of CM in other industries.

## 8. Opportunities and Value-Added Applications

Analyses of the chemical and physical properties indicate a great opportunity for other potential applications and the further value addition of the product. As the world moves towards a more sustainable agri-production system, the growing interest in fully utilizing agricultural wastes, especially those produced in bulk, is a major concern. For instance, there is an increasing trend in the use of oil cakes to produce substrates that will serve as carbon and nitrogen source in manufacturing biochemicals, including enzymes and antibiotics, through fermentation [71]. Aside from the extensive studies being conducted to optimize the use of copra meal as an alternative feed ingredient, various researchers are now seeking to explore the utilization of copra mannan for the production of prebiotic manno-oligosaccharides (MOS) for food and feed applications [72,73,74,75,76,77,78]. Humans, similar to animals, cannot readily digest copra meal due to its high mannan content. However, the β-mannanase enzyme can break down the complex mannan polysaccharide into MOS [75]. Various studies have investigated copra meal hydrolysate (CMH), created by hydrolyzing defatted copra meal with food-grade β-mannanase, as a potential prebiotic to support the growth of beneficial gut bacteria and enhance gut health [75,76]. The consumption of CMH was found to improve bowel movements and provide relief from bloating symptoms [76]. Furthermore, studies have shown that CMH promotes the growth of beneficial microbes in the human intestinal microflora, while reducing the levels of pathogenic bacteria such as *Escherichia coli* [75,76,77,78]. Moreover, MOS can be potentially used to replace antibiotic growth promoters and as a mycotoxin and cholesterol binder in poultry diets [74]. Mannans are also widely used in other food industry applications as thickening, stabilizing, and gelling agents, but have not yet been commercially pursued in the context of copra mannans.

The potential bioconvertibility of copra meal as a substrate for bioethanol production was tested by Antia et al. [79] at varying concentrations. Since CM contains a significant amount of mannan polysaccharides, the samples were pretreated with alkali and acids and through autohydrolysis and additional enzymatic hydrolysis prior to fermentation. The experimental setup led to a viable ethanol yield as high as 0.47 g/g at a 20% (*w*/*v*) substrate concentration after 48 h of continuous saccharification and fermentation. This result was comparable to the theoretical yield obtained from other lignocellulosic substrates at about 0.51 g/g. However, the economic impact of the additional steps of pretreatment prior to the fermentation proper, which could have detrimental effects on the overall technical and economic feasibility of the process, has to be further analyzed, especially for larger-scale production.

Another area that has received significant attention in the past few years is the use of waste biomass for the production of biofuels. Despite its high energy value, there is limited available literature exploring the potential use of CM as an energy source, although other coconut-derived products, such as the husk, shell, frond, fiber, and pulp, have been widely explored in energy generation, as in the study of Azeta et al. [80]. Among the limited existing research performed in this field is that of Szyszlak-Bargłowicz et al. [62], which evaluated the possibility of incorporating copra meal as an additive in the production of giant miscanthus (*Miscanthus giganteus*) biomass pellets. Varying levels of CM ranging from 10 to 100% were added to the biomass blend and the resulting pellets were analyzed in terms of the energetic and physico-chemical properties. The results revealed that the increasing levels of CM in the mix resulted in higher heating values and lower energy consumption in the pelleting process, thereby increasing the energy efficiency (EE) and energy yield (EY) of the miscanthus pellets. However, the CM additives had detrimental effects on the mechanical durability and pellet density, which are directly correlated to the heating applications and logistical risks, respectively. The experimental study was also limited regarding the energetic properties and was not extended to the suitability of the resulting pellets to serve as biofuel; hence, the further examination of CM’s actual performance as a biofuel component must be undertaken. Nevertheless, the improvements in the densification process and the properties of biomass pellets provide an opportunity for the potential application of CM as an alternative energy source and they could further be tested in other types of biomass.

Due to the structural stability, high absorption capacity, and porous structure of various coconut parts and biomass, their potential use in the adsorption of toxic pollutants such as heavy metals, industrial dyes, pharmaceutical, and organic contaminants has also been explored, and the initial findings indicate promising results. The most commonly used coconut parts in the development of low-cost bio-adsorbents are the coconut shell, husk, and coir, in their native or modified forms [81]. As more research studies focus on the development of coconut biomass into environmentally friendly and low-cost bio-adsorbents, a couple of researchers have tested the possibility of utilizing raw copra and copra meal for this purpose. CM has been found to be easily modified into bio-adsorbents due to its soft texture attributed to its high mannan content [82]. The study conducted by Lee et al. [83] revealed promising results on the use of copra for cadmium adsorption from water, demonstrating a maximum adsorption capacity of 1.092 mg/g and efficient removal for up to seven consecutive cycles of adsorption and desorption. Similar studies on the use of raw copra for the removal of heavy metals were performed by Lee and Sim [84] for Cd, Cr, and Ni from aqueous solutions, while that of Simarani et al. [85] tested the feasibility of using grated copra biomass for the removal of dye contaminants such as methylene blue. For copra meal itself, only one published study, performed by Saleem et al. [82], has tested the potential application of CM biomass in divalent nickel ion removal from aqueous solutions, revealing a maximum monolayer NI (II) capacity of 3.77 mg/g. The experimental data fitted the Langmuir isotherm model, which indicates the excellent potential of using a CCM biosorbent to economically and sustainably remove Ni (II) contaminants from wastewater.

The potential replacement of single-use synthetic polymer-based plastics with cheap and biodegradable thermoplastics made from coconut meal has been recently studied by Reddy et al. [86]. Since copra meal, just like other oil meals, is non-thermoplastic in nature, varying types and ratios of plasticizers were added to enable thermal processing before the meal was subjected to compression molding and rendered into biofilms. The use of 5% glycerol produced films with tensile strength of 2.4 MPa and a modulus of 305 MPa, comparable with paper-based plastics. On the other hand, the further addition of glycerol was found to lower these mechanical parameters due to the inherent hydrophilic characteristic of the substance. Different oils, including coconut, peanut, and cashew nut shell liquid, were also tested as plasticizers. However, the results varied considerably depending on the thermoplastic and hydrophilic properties of the oil used, with coconut oil providing optimal stability and mechanical properties even at higher humidities. Citric acid was also tested for the crosslinking of the bioproduct and the results showed excellent retention, suggesting its possible application for aqueous and semi-aqueous items. Overall, the possible conversion of copra meal into biothermoplastics shows positive and cost-effective results with just the compression molding technique, without the need for high-end chemical modifications, although this is another area for exploration in this field. The study also provides evidence of the ease of molding copra meal into any shape and form, so that it can be used for food and non-food handling and packaging, since it is edible and biodegradable and exhibits good antimicrobial resistance. Note, however, that the aflatoxin contamination of raw copra meal is among the major concerns, as for any of its applications, and the quality of the input resources must be ensured should it be used for this purpose [39,87].

In terms of optimizing the use of CM as an alternative animal feed ingredient through nutritional improvement, efforts are being shifted towards large-scale production and the use of fermented copra meal given its positive experimental results, as proven in various studies. In 2017, the Philippines began the establishment of a pilot plant facility with a 1 MT production capacity for fermented copra meal, called Protein-Enriched Copra Meal (PECM) [88], followed by the recent commercialization of the product, funded through the 2022 annual budget of the Department of Agriculture of the Philippines. Further study can consider the effects of an industrial setup on the quality and economics of fermented copra meal. As Kraikaew et al. [89] observed, increasing the production scale from a 200 g laboratory scale to a 10 kg industrial capacity within a simultaneous saccharification and fermentation setup of copra meal resulted in higher protein content but decreased the yield, attributed to the mixing system and the more efficient oxygen transfer rate in the larger fermenter tank.

## 9. Conclusions

With coconut oil production making up a significant portion of the coconut industry, there is a huge stream of byproducts, particularly copra meal, directly derived from the process. Copra meal has been widely studied and partially incorporated as an alternative feed ingredient, but there are still challenges in its utilization, resulting in the bulk of the volume proceeding downstream as agricultural waste. Understanding the inherent characteristics and composition of this product is the first step in exploring and opening up opportunities for value-added applications. As outlined in this paper, there are gaps in the characterization of copra meal, since chemical and nutritional analyses have been limited in the context of using it as animal feed, while comprehensive information on its physical properties is still lacking.

Identifying the prospects and value-adding opportunities for copra meal could pave the way for a more competitive coconut sector, making the industry more profitable for coconut farmers and copra processors, especially in tropical countries, where the majority of production is found. Improvements in the use of copra meal could also contribute to the goal of achieving sustainable agri-production systems in the long run by reducing the amount of agricultural biomass being wasted and instead converting it into low-cost, environmentally friendly products.

However, further analyses are needed to cover other byproducts along the coconut value chain and the wide variations in the quality of the resulting copra meal based on the processes and technologies employed. Although limited, there have been initial studies that have looked into these possibilities, some of which have been discussed in this paper, but other important and emerging applications, such as in human food and industrial products, or new developments in the methods of component extraction and conversion, should also be investigated. There is still much more to consider in order to paint a complete picture of the usability potential of copra meal, elevating its potential to that of other coconut byproducts and co-products and oil cakes from conventional sources.

## Figures and Tables

**Table 1:** Coconut oil importation [27].

Importing Country/Region	Volume (‘000 MT)		Percent Change (%)
	2021	2022	
European Union (EU)	616	691	12.2%
USA	468	535	14.3%
Malaysia	225	360	60.0%
China	174	219	25.9%
Other Countries	516	542	5.0%
World	1999	2347	17.4%

**Table 4:** Physical properties of copra meal.

Property	Reported Values	References
Bulk Density (g/cm^3^)	0.49–0.56	[7,10,11,64]
Water Holding Capacity (g water/g feed)	4.14–4.18	[7,10,11,64]

## Data Availability

Data sharing is not applicable as no new data were created or analyzed in this study.
